# Small Molecule Targeting of Protein–Protein Interactions through Allosteric Modulation of Dynamics

**DOI:** 10.3390/molecules200916435

**Published:** 2015-09-10

**Authors:** Benjamin P. Cossins, Alastair D. G. Lawson

**Affiliations:** Computer-Aided Drug Design and Structural Biology, UCB, 216 Bath Road, Slough, SL1 3WE, UK; E-Mail: alastair.lawson@ucb.com

**Keywords:** protein–protein interaction, allosteric, protein dynamics, small molecule

## Abstract

The protein–protein interaction (PPI) target class is particularly challenging, but offers potential for “first in class” therapies. Most known PPI small molecules are orthosteric inhibitors but many PPI sites may be fundamentally intractable to this approach. One potential alternative is to consider more attractive, remote small molecule pockets; however, on the whole, allostery is poorly understood and difficult to discover and develop. Here we review the literature in order to understand the basis for allostery, especially as it can apply to PPIs. We suggest that the upfront generation of sophisticated and experimentally validated dynamic models of target proteins can aid in target choice and strategy for allosteric intervention to produce the required functional effect.

## 1. Introduction

Selection of small molecule targets has a crucial bearing on the success or failure of individual projects [1,2,3]. Genetic and/or clinical validation, often with a biological therapy, can help to establish confidence in a target and reduce biological risk, and, more recently, the concept of structure-based druggability or ligandability has become an important complementary factor. Protein–protein interactions (PPI), which were previously considered to be undruggable, are now thought of as challenging but tractable [4,5,6]. In this review we consider ways in which the study of protein dynamics at a very early stage of target selection may further reduce risk in drug discovery and lead to opportunities for ‘first in class’ therapy with small molecules [7].

The PPI target class is defined as any small molecule which directly modulates the interaction between two or more proteins. This excludes situations where a small molecule modulates a naturally occurring allosteric signalling pathway seen in targets such as G-protein-coupled receptors (GPCRs). Hence, small molecule binding sites for PPIs are not evolved for binding small molecules and directly modulate the PPI without using a naturally occurring allosteric signal. The PPI network or interactome has been mapped through a high-throughput yeast two-hybrid approach to be ~130,000 binary interactions [8], so there is great potential for new therapeutic interventions which inhibit or stabilise PPIs.

We introduce a new term “functionability”, meaning the ease with which a functional compound can be found for a biological drug target. This is distinguished from “ligandability” which focuses on the discovery of small molecules which bind. The idea of functionability is important when considering allosteric mechanisms, as here binding and functional modulations can be separated. When we are willing to take on development through allosteric mechanisms, a thorough and detailed analysis of protein target dynamics at an early stage can enable informed decision making about the functionabilty of targets and any discovered remote pockets. This means producing a validated dynamic, thermodynamic and possibly kinetic model including any conformational changes and searching for local and long range dynamic correlations.

## 2. Prevalence and Types of PPI Modulation

Three databases of small molecule PPIs have been compiled in recent years which contain both inhibitors and stabilisers [9,10,11]. These databases suggest there are currently around 50 PPI targets with small molecule modulators in the public domain, 25 of these with PDB entries.

Orthosteric PPI inhibitors are characterised by binding sites which are generally more challenging than those evolved for small molecule binding (enzyme pockets) owing to their large flat nature [4,5]. For this reason PPI inhibitor programmes often struggle to improve on the micromolar binding affinity range, and small molecule inhibitors are mostly larger than average drug molecular weights [12], with alternatives such as macrocycles or peptides being of interest [13,14,15,16,17].

PPI stabilisation may be less challenging as the interface of two bound proteins tends to produce buried cavities at the margins, with similarities to enzyme pockets [18]. In practice small molecules which bind to cavities formed from two binding partners, either stabilisers or inhibitors are still less common than orthosteric inhibitors which bind to one partner. This may be related to the increased difficulty of producing crystal structures of PPI complexes.

An alternative situation is where small molecule binding sites are remote to the PPI site with an allosteric mechanism of modulation. In addition to offering more viable alternatives to the unattractive PPI sites, allosteric small molecules offer other advantages. Allostery allows for the possibility of more control over function in terms of partial inhibition, dose dependency and agonism [19,20]. Also allosteric sites may offer more selectivity, as they are generally more varied between related proteins however, this increased variation may also lead to a higher rate of tolerated mutations. Additionally, some allosteric pockets offer the possibility of causing dissociation of a pre-bound PPI complex which may be attractive in some cases. Despite these advantages, allosteric PPIs are rare compared to those with an orthosteric mechanism; only 2.4% of the active compounds listed in the TIMBAL database are labelled as allosteric in ChEMBL [21]. This is probably related to the difficulty of discovery or generating function from remote pockets. 

## 3. Protein Dynamics and the Nature of Allostery

Concepts of allostery are often based around particular directions of study such as energy surfaces, conformational change, ensembles or communication pathways/networks [22,23,24,25], however, a unified view is possible and correct [26,27]. Allostery is mediated through modification to the dynamic ensemble and energy surface of the target protein, but it is not necessarily easy to detect in terms of traditional experimental structural data [28]. The practical study of protein dynamics has moved forward significantly in recent years in some part owing to the combination of advanced molecular simulation, crystallography and spectroscopy techniques like NMR. The emerging picture of protein behaviour has increasing complexity with many proteins having multiple conformational states, even those previously thought to be relatively rigid [29,30], and complex networks of correlated functional units [31,32]. Additionally, small molecule ligands bind to PPI binding site configurations which are not observed in crystal structures of the apo protein, suggesting that PPI sites can have significant dynamics [4,33,34].

Allosteric modulation is sometimes explained in terms of multiple kinetically separated conformations where a modulator stabilises one state over the other(s) [27]. Searching for these PPI incompetent conformations is difficult, as they can be lightly populated and hidden behind thermodynamic barriers making them difficult to characterise. An alternative conformational allosteric mechanism induces new, non-functional, conformations which are not populated naturally [35].

Even without multiple kinetically distinct conformations, allosteric modulation was predicted to be possible [36]. Fundamentally this allostery is mediated through remote regions of a protein having correlated fluctuations. Simple models suggest long range correlation requires an inherent elastic inhomogeneity [37]. Proteins are naturally organised in this way with units of residues which are correlated and differing levels of correlation between each of these units [31,32]. Hence, long range correlations are likely common. Computational methods for evaluating normal modes have been widely used for many years [38] to describe the low-frequency motions of a protein within a single kinetic basin. Modification of local stiffness can affect these low-frequency modes and may allow a clear understanding of long range allostery [39,40]. These models provide theoretical detail to studies of well-known proteins demonstrating allosteric disruption of remote binding sites through changes in configurationally entropy [41,42]. 

## 4. Types of Allosteric PPI

Allostery is classified in various ways [23]; here we seek to classify in a way which explains how we can impact the discovery of functional chemical matter for PPI drug discovery through understanding protein dynamics.

### 4.1. Re-Balance Naturally Existing Conformational Change

Small molecules can stabilise alternative conformations which can inhibit a PPI. These cases are not always simple to understand; especially when the inhibitory conformation is populated very lightly and experimental structural data are not available.

Lymphocyte function-associated antigen-1 (LFA-1) is an example of this modulated conformational change [43,44]. There is currently a compound in clinical trials which is able to inhibit allosterically the interaction of LFA-1 with ICAM-1 [45]. The more active and less active conformational states have been trapped out using disulphide bonds introduced after a study of available crystal structures [44] giving a clear mechanism of conformational change for the associated compounds. In cases where crystallography of the relevant conformations is not available, a rational approach becomes more difficult. 

### 4.2. Modulation of Dynamics without Conformational Change

An early example of this allosteric disruption within the same conformation is the inhibition of dimerization of inducible nitric oxide synthase (iNOS) [46]. A focused library of compounds based on an understanding of haem ligands selective for iNOS over endothelial NOS was used to find dimerization inhibitors through a cell assay. Crystal structures with and without the best of these inhibitors clearly show the allosteric mechanism behind the disruption of the secondary structure of the PPI.

The PPI between Runx1 and CBF-beta is another interesting example of allosteric disruption within the same conformation. A virtual screening effort using 20 conformers of CBF-beta generated 35 compounds of which four showed significant chemical shift changes in 2D HSQC spectra of CBF-beta. The chemical shifts were assigned to a site on a beta-sheet which is remote from the PPI site [47]. The structural mechanism for these allosteric inhibitors seems to be through the propagation of induced changes in side-chain dynamics from the allosteric pocket to the PPI site. 

In addition to these two types of allosteric PPI there are a series of examples related to protein oligomerisation [48]. One particularly interesting example, which has been in clinical use for some time, is that of microtubule-stabilising agents (MSA) such as Taxol. MSAs are anti-cancer agents which modulate the structure and dynamics of tubulin such that extra lateral interactions between oligomers are produced promoting microtubule assembly [49,50].

## 5. Practicalities of Finding and Optimising Chemical Matter

When screening for PPI modulators, ultimately there is one important question: what is the functional effect? Consequently many approaches start with a functional screen, generally through detection of a change in PPI binding or target stability [6,51]. Others start by searching for binding and then check for a functional effect, as sometimes it pays to understand binding and binding sites even in the absence of detectable functional hits. Detecting weak modulation of a PPI can be difficult, especially for fragment screening, owing to variation in the nature of the protein partners and the generally weak binding and functional signal which fragments offer [5,52,53,54]. 

Allosteric interventions have the added difficulty that function/inhibition is not necessarily correlated to the binding affinity of the small molecule and must, to some degree, be optimised separately. It is our ability to understand this that will enable us to make extensive use of remote binders which have weak or no function. An enrichment of allosteric small molecules is something seen in other target classes where orthostery is difficult such as class C GPCRs [20,55]. In future we may well find an increasing number of allosteric modulators of PPIs reported.

## 6. Directions for Future Success with Allosteric PPIs

The main difficulty with allosteric PPIs is the initial generation and optimisation of functionally active compounds. One strategy to remedy this is the application of new screening technologies, which may provide more functional hits, both orthosteric and allosteric. Hit rates for PPIs are low in traditional HTS and fragment-based approaches, but some very large libraries have demonstrated greater than expected numbers of functional hits even in the most difficult cases [56,57]. Currently there are very few thorough studies systematically comparing hit rates for PPI targets, and there are many complicating factors of applicability for different approaches [58]. For instance the yoctoreactor and binder trap enrichment approach has compounds tethered to a large DNA tag which would make reaching buried binding sites difficult and may modify binding in other cases. 

A possibly cost effective route is to follow a more rational approach to finding chemical matter. Hence, the emerging understanding of how to probe protein dynamics and allosteric mechanisms can be used to evaluate the allosteric functionability of targets. This would involve searching for alternative conformations which inhibit PPIs or evaluating the potential for dynamic correlations, which can modulate the PPI site. For cases in which allosteric functionability is favourable, this naturally leads to a well-conceived rational strategy for identifying chemical matter and directing its further development.

An alternative conformation would need to be sufficiently thermodynamically stable, such that modulation with a small molecule could cause it to compete with the naturally dominant conformation. The combination of molecular dynamics (MD) and experimental validation/exploration can provide valuable structural and thermodynamic information in these difficult cases [59,60,61]. One drawback of MD is that of limited time-scales. This can make scaling large thermodynamic barriers very difficult and cause useful conformations to remain hidden. In some cases these problems can be overcome through using enhanced MD which uses some form of driver function [62,63], although these approaches often require detailed knowledge of the difficult barriers to be overcome. Therefore, an initial exploration with experimental methods, such as capture with antibodies, can provide the motivation to search in particular directions with MD [64]. An additional draw-back of MD is that of potential force-field inaccuracy. To provide confidence in discovered conformations, generally applicable and sensitive experimental solution approaches are required. Interesting options for validation of simulation include double electron-electron resonance spectroscopy (DEER) [65], and mutation studies [66]. Despite these advances it is clear that more advanced approaches are still required, as even with the advent of millisecond simulations [67,68] and enhanced MD [69,70] hidden yet druggable conformations are still difficult to find.

For allostery within one kinetic conformation, the key is to gain exquisite understanding of the collective motions of the protein target. Understanding a protein target through blocks of residues with correlated dynamics rather than secondary structure or evolutionary conservation is an important concept emerging in the literature [31,32]. These blocks or communities of residues are correlated with each other to differing degrees and can be functionally annotated. The reliable evaluation of these correlations has become possible owing to the combination and development of relationships from mutual information theory and statistical mechanics which allow the detection of correlation signals in relatively short calculations [71]. Experimental investigation of protein collective motions is possible through NMR relaxation dispersion approaches [72] and offers great potential synergy with simulation approaches. Armed with these correlation maps it may be possible to produce higher quality simplified models with which we can rapidly evaluate the likelihood of useful modulations to long range modes from binders in particular surface positions [73]. This would enable rapid evaluation of functionability when searching for hidden pockets with MD [61,74] or decisions about weak or non-functional remote binders, discovered through experimental screening, to be made. In order to validate these models, the tethering of reactive probes [75,76,77] can be used to confirm functional binding sites found computationally or to scan a protein for functional sites. If these approaches can be successfully coupled with *de-novo* design or virtual screening, they will offer a powerful route to novel hits in novel pockets.

## 7. Conclusions 

PPI drug discovery is considered one of the most difficult of small molecule target classes in discovery research owing to the generally unsuitable orthosteric binding sites. More attractive allosteric pockets may offer a useful alternative, but currently a lack of understanding of these mechanisms and routine methodologies to take advantage of them, mean that there are still very few examples. The up-front development of sophisticated models of protein dynamics and correlations offer a more rational and potentially productive route to understanding functionability and finding chemical matter in these difficult cases. Finally, an improved understanding of protein dynamics and correlations is also helping us to understand how protein conformations have evolved [78].
